# False Novelty in Antibacterial Natural-Product Discovery: Rediscovery, Structural Misassignment, and a Checkpoint Framework for Early Verification

**DOI:** 10.3390/ph19081182

**Published:** 2026-07-28

**Authors:** Gadah Abdulaziz Al-Hamoud

**Affiliations:** Department of Pharmacognosy, College of Pharmacy, King Saud University, Riyadh 11451, Saudi Arabia; galhamoud@ksu.edu.sa

**Keywords:** antibacterial discovery, natural products, dereplication, structural misassignment, structure revision, computational NMR, DP4+, molecular networking

## Abstract

Antimicrobial resistance is outpacing antibacterial innovation, yet most explanations for the shrinking pipeline emphasize downstream economic and regulatory attrition. This review identifies an underexamined upstream bottleneck: the unreliable recognition of genuine chemical novelty when a natural-product hit is first encountered. It advances a unifying framework—false novelty—that treats two conventionally separate failures—the rediscovery of known metabolites and the structural misassignment of genuinely new ones—as coordinate manifestations of a single diagnostic failure, an integration rarely made explicit. Eleven independently verified cases from 2019 to 2025, spanning three rediscoveries and eight misassignments, demonstrate that both failures persist under modern instrumentation. Their persistence, therefore, appears to reflect inconsistent workflow adoption more than a lack of technical capability, since dereplication and computational structure-verification tools mature enough to intercept both are already available. The review translates this diagnosis into a resource-conscious, three-checkpoint verification workflow that matches each failure mode to an appropriate remedy at the discovery stage, where correction is cheapest. Recognizing novelty earlier offers a tractable route to conserving scarce discovery-stage resources.

## 1. Introduction

Antimicrobial resistance (AMR) continues to widen the gap between clinical need and therapeutic supply. According to WHO’s 2025 [1] analysis of the global antibacterial pipeline, only 90 antibacterial agents remain in clinical development, down from 97 in 2023. Of these, just 15 qualify as genuinely innovative, and only 5 are active against WHO critical-priority pathogens such as carbapenem-resistant *Acinetobacter baumannii*. Between 1940 and 1962, more than 20 new antibiotic classes were successfully marketed [2]. Innovation has since slowed sharply: the most recent genuinely new class of antibiotics was discovered during the 1980s, and most agents approved thereafter were derived from already known chemical scaffolds [3]. WHO now frames this situation as a “dual crisis of scarcity and innovation” [1].

Existing explanations for this stagnation converge on economic, regulatory, and biological factors. Weak market incentives discourage sustained investment in new antibacterial therapies [3]. Achieving selective toxicity against Gram-negative pathogens remains a persistent technical obstacle, and resistance mechanisms have spread across nearly all legacy drug classes, eroding the utility of incremental analog development [2]. These accounts are well documented and important, but they primarily describe late-stage attrition, in which candidate molecules fail during development, regulatory review, or commercialization.

Comparatively little attention has been paid to a bottleneck occurring earlier in the discovery process: the reliability with which a candidate natural-product hit is recognized as genuinely novel before committing resources to isolation and structural elucidation [4]. This bottleneck reflects a deeper paradox. Most microbial biosynthetic potential remains chemically unexpressed under standard cultivation, yet screening programs continue to converge on the same narrow chemical space. Two failure modes follow from this pattern. First, natural-product antibiotic discovery programs have long acknowledged a rediscovery problem, in which bioassay-guided screening repeatedly reisolates already-known metabolites [4,5]. The countermeasure, dereplication—the early identification of known compounds—was so named in the 1980 CRC Handbook of Antibiotic Compounds [5]. Second, structural misassignment occurs when incomplete or ambiguous spectroscopic evidence leads to the publication of an incorrect structure for a new chemical entity [6]. Such misassigned structures are often revised only years later through total synthesis or advanced spectroscopic reanalysis [7]. Both modes generate what this review terms false novelty: a hit that appears new by the standards of the tools and databases available at the time but does not withstand rigorous downstream scrutiny [4,6].

This review argues that early failure in novelty recognition is an underexamined upstream source of inefficiency in the natural-product antibiotic pipeline [4,6]. It manifests as rediscovery and structural misassignment and is a plausible, though not yet quantified, contributor to pipeline stagnation. This upstream barrier is distinct from and complementary to the economic and regulatory factors more commonly discussed in the AMR policy literature [1,3]. It is also distinct from a third, prominent research direction: AI-driven efforts to expand the searchable chemical space of synthetic and repurposed compound libraries. This direction is exemplified by deep learning models that predict antibacterial activity from molecular structure and have identified novel candidates, such as halicin [8]. That direction addresses how many candidates enter the pipeline; this review addresses whether the candidates that do enter—specifically those from natural sources—are reliably recognized as novel once found. Comparatively little systematic attention has been paid to whether current dereplication and spectroscopic practices are adequate to make that distinction at the point where natural-product discovery actually begins: the crude extract or early fraction. Crucially, the technical means to detect both failure modes early are already mature and increasingly accessible. Computational NMR verification tools such as DP4+ are now user-friendly enough for non-experts and can flag misassignments before resource-intensive synthesis is undertaken. The persistence of false novelty therefore appears to reflect a gap in workflow adoption more than a lack of technical capability [7]. This is a systemic gap in the routine integration of available methods.

The principal contribution of this review is threefold. First, it unifies rediscovery and structural misassignment—two failure modes conventionally treated in isolation—under a single explanatory construct. Both phenomena are individually well documented [4,6]. This review treats them explicitly as coordinate manifestations of one diagnostic failure rather than as unrelated problems. To this end, it adopts the term false novelty as a unifying analytical framework, not as a newly coined label. The term is used here strictly to denote apparent chemical novelty that fails later verification (through dereplication or structural re-analysis). It does not imply deliberate error, misconduct, or intent to mislead on the part of the original investigators. Second, it documents that both failure modes persist despite the availability of mature technical remedies, locating the problem in routine practice rather than in the underlying tools. The evidence comprises eleven independently verified cases spanning 2019 to 2025. These fall into three confirmed rediscoveries (cottoquinazoline F, triculamin/alboverticillin, and leepeptin/chaxapeptin), eight structural misassignments (myxovalargin, chelocardin/amidochelocardin, serratiomycin, evybactin, bacilotetrin A, relacidine A/B, lydiamycin A, and halisphingosine A), one of which—relacidine A/B—was an originally unassigned stereochemistry rather than an outright error. Third, it translates this diagnosis into three concrete, immediately actionable workflow adjustments for natural-product antibacterial discovery programs, detailed in the section on an integrated framework below.

The review proceeds by first situating novelty-recognition failure within AMR pipeline attrition. It then examines documented cases of rediscovery and misassignment. Finally, it evaluates current dereplication and spectroscopic methodologies in light of this failure mode and proposes a verification framework before closing with a discussion of limitations.

## 2. Search Strategy and Case Selection

This manuscript is a critical narrative review, and its design is deliberate: the central contribution is conceptual integration and interpretation. Cases were identified through structured searches of PubMed, Web of Science, and the Dictionary of Natural Products. A single term set was applied in PubMed and Web of Science to ensure a reproducible search. Forward and backward citation tracking from established structure-revision sources, including Yoo et al. [6] and Beduru and Kutateladze [9], extended this coverage. The searches were conducted between 2025 and 2026, whereas the 2019–2025 window denotes the targeted publication range for the retained cases. Table 1 documents the databases, search terms, and publication range, and Figure S1 (Supplementary Materials) summarizes the case-selection flow, making the selection process fully transparent and traceable.

This lower bound is principled: by 2019, high-resolution mass spectrometry and modern computational NMR verification had become routine, so every retained case arose under current analytical conditions rather than the sparse data of earlier eras. A case was retained only when novelty-recognition failure—rediscovery or structural misassignment—was documented in an antibacterial natural product and independently verified in the primary literature by total synthesis, X-ray diffraction, or advanced computational reassignment. This confinement to the modern-instrument era is the analytical core of the argument: it demonstrates that novelty-recognition failure is not a historical artifact of outdated instrumentation but a persistent challenge that recurs even when state-of-the-art tools are readily at hand.

The review confines its scope to antibacterial activity rather than the broader antimicrobial spectrum. This reflects the clinical crisis motivating the analysis: the pipeline documented by the World Health Organization [1] is specifically bacterial, and a single target preserves the mechanistic homogeneity that makes a recurrent failure pattern identifiable. Eligibility therefore turned on antibacterial activity itself, irrespective of whether the producing organism was bacterial, fungal, or invertebrate.

The final set of eleven cases (Table 2 and Table 3) was purposively selected for its analytical relevance, structural diversity, and recency. A focused set of rigorously verified recent instances illuminates the failure mode far more sharply than an undifferentiated census would. These eleven cases stand as well-verified, decisive exemplars of an underexamined problem; the implications of this design for generalizability are examined in Section 8 below.

## 3. Background: Positioning Novelty-Recognition Failure Within AMR Pipeline Attrition

The dominant response to the historical decline in new antibiotic classes, articulated by Coates et al. [2], is to rebuild a 1940s-scale discovery effort through financing, legislation, and infrastructure. That prescription rests on an unstated assumption: that natural-product discovery is a sound process awaiting adequate resources. A separate strand of literature challenges precisely that assumption, showing that the process itself is compromised at the discovery stage.

Genilloud [4] makes the challenge explicit, identifying the ‘rediscovery problem of old known molecules’ as a methodological limitation that operates alongside, and is not reducible to, economic disincentives. It is a persistent failure at the discovery stage that consumes resources even where investment exists. Baranova et al. [5] trace this failure to its source, identifying it as one of three inherent limitations of the phenotypic “Waksman platform” that produced the mid-century boom. Broad-spectrum activity screens systematically favor toxic or well-known compounds over selectively active novel ones. The screening paradigm invoked as the solution is therefore itself a source of re-isolation. Scaling up the historical approach, as Coates et al. [2] recommend, would reproduce the rediscovery problem at greater volume rather than resolve it.

Lin et al. [10] reisolated cottoquinazolines E–G from *Neosartorya fischeri*, reinvestigated their absolute configuration, and found that cottoquinazoline F retained activity against four priority pathogens (ESBL-*E. coli* among them). The activity reentered the literature only as a byproduct of that stereochemical work, not through deliberate dereplication.

Andersen et al. [11] and Gomez-Escribano et al. [12] extend the pattern (see Table 2). In each, a compound assumed novel at isolation was shown, downstream, to duplicate an earlier one—after full characterization rather than before it. These three cases share a single structural signature: novelty is granted by default at isolation and withdrawn only downstream, showing that false novelty is not a series of isolated mistakes but a recurring failure at the recognition step.

**Table 2:** Verified rediscovery cases. Extended version in Supplementary Table S1.

Compound Pair	Original/Later Identity	Gap	How Identity Was Established
Cottoquinazoline [10]	Strain first reported 2015; reisolated 2020 during an unrelated stereochemical study	~5 yr	Reisolation + configurational reinvestigation; activity vs. ESBL *E. coli*, *A. baumannii*, *P. aeruginosa*, *E. faecalis* (MIC 8–32 µg/mL) recognized only incidentally
Triculamin = Alboverticillin [11]	Alboverticillin (1958) and triculamin (1967) were shown to be one lasso peptide	~55 yr	2D NMR + genome sequencing during biosynthesis investigation
Leepeptin/Chaxapeptin [12]	Of three lasso peptides from *S. leeuwenhoekii* C34^T^, one novel (leepeptin); the other two matched known peptides—chaxapeptin (2015, Lp3) and a citrulassin-family peptide (Lp1)	~4 yr	LC-HRMS + NMR comparison within the same heterologous-expression study

Well-documented recent rediscovery cases remain less common in the literature than the misassignment cases cataloged below. Two readings are possible: that rediscovery is genuinely infrequent, or that it is under-reported. The publication incentive favors the latter, since a confirmed rediscovery is less publishable than a novel or corrected structure and is less likely to be written up. Consistent with the long-recognized tendency to treat re-isolation as a nuisance [13], the reported count may therefore represent a lower bound rather than a full measure, though this remains a hypothesis rather than an established conclusion.

A second, mechanistically distinct failure mode compounds this picture. Yoo et al. [6] document 21 natural-product structures, several of which were reported to be bioactive, published as novel and later revised after an NMR misinterpretation was identified. Here, the compounds were genuinely absent from prior literature, yet the tools used to establish novelty generated a false positive for novelty itself. Yoo et al. [6] attribute this persistence, despite continuous gains in instrumentation, to interpretive rather than technical limitations.

Together, these cases illustrate a bottleneck that the economic-attrition account does not capture. Genilloud [4] and Baranova et al. [5] identify rediscovery as a methodological, output-limiting failure; Yoo et al. [6] add misassignment, which operates even when a compound is genuinely new. The economic account explains why fewer resources reach discovery, not why a meaningful share of those that do are spent re-isolating known compounds or validating structures that later prove wrong. That gap is what this review addresses.

Tulp and Bohlin [13] framed rediscovery as ambivalent two decades ago, noting that re-isolation dismissed as a nuisance can equally signal a conserved, pharmacologically validated scaffold. As the pipeline has narrowed to 90 agents, of which only 15 are innovative, and 5 are active against critical-priority pathogens [1], scarce discovery-stage resources cannot absorb avoidable re-isolation. The fully capitalized cost of a single approved drug is roughly US $2.6 billion, inclusive of failed candidates [14]. Against that figure, effort spent characterizing a compound that dereplication could have excluded at the extract stage is a measurable loss at the stage where the pipeline can least afford it.

Not every researcher accepts that recognition failure meaningfully constrains output. Skinnider and Magarvey [15] argued that a statistical reanalysis of structural data reveals increasing rather than declining chemical diversity over time. Pye et al. [16] replied that their own analysis had been misread and that the discovery rate of genuinely new molecular architectures remains robust. That exchange concerns the aggregate trend across literature. The present review addresses a different level: the reliability of the recognition step for any single hit, which neither side of that debate examined. The individual rediscovery and misassignment events documented here therefore stand outside that dispute rather than against either position in it.

## 4. Early Novelty-Recognition Failure: The Persistence of Misassignment

The temporal distribution of misassignment cases is informative. Yoo et al. [6] documented 21 revised structures accumulated through the mid-2010s; in 13 of them, the error lay not in constitution but in stereochemistry, since NOESY and ROESY data do not resolve absolute configuration. A decade later, Beduru and Kutateladze [9] curated a comparable set spanning 2018 to 2025—a period in which NMR instrumentation, computational power, and machine-learning-augmented DFT methods, including their DU8ML approach, advanced substantially. The critical point is not that misassignments exist in both periods, but that they continue to accumulate despite these tool-level improvements. Were misassignment primarily an instrumentation problem, the 2018–2025 cohort should be markedly smaller or confined to structural outliers. It is not.

This continuity across a decade of instrumentation progress supports a stronger claim than “tools are imperfect.” The recurring failure appears to lie in the interpretive workflow connecting spectral data to a structural hypothesis, not merely in the resolution of the spectra themselves. de Albuquerque et al. [7] make this workflow problem explicit, asking whether the field is still “chasing molecules that were never there.” Their 2019–2023 survey found misassignments persisting despite the routine availability of quantum-chemical NMR verification tools. Their answer is functionally yes: misassignment remains a recurring theme in modern literature. Comprehensive structural verification is treated as optional rather than routine, given its time and expertise costs relative to a publication deadline [7].

The hexacyclinol case, revisited by de Albuquerque et al. [7], is instructive because misassignment survived an initial total-synthesis effort. An early total synthesis built on the original structure failed to reproduce the natural product’s spectroscopic data. Rychnovsky’s [17] independent computational NMR reanalysis then established the correct diepoxide structure, which Porco et al. [18] confirmed by total synthesis. This sequence—proposal, full synthetic commitment, and only then discovery of misassignment—illustrates how misassignment can waste synthetic effort that verification would have spared. Applied before synthesis, computational NMR reanalysis would have made that misdirected work avoidable.

This failure mode is neither unique to antibacterials nor new to the past two decades. Nicolaou and Snyder [19] surveyed a much longer history of misassigned natural products across chemistry, concluding that chemical synthesis is the definitive arbiter of structure whenever spectroscopic evidence alone proves ambiguous. Computational NMR methods now serve the same diagnostic role as in hexacyclinol [17] and later catalogs [6,9]. What is specific to the antibacterial context is not the error mechanism but its consequence. A misassigned structure propagates into an already scarce, closely watched pipeline [1], where the margin of viable candidates is far smaller than in natural-product chemistry at large.

The scale of the problem confirms it is more than anecdotal. A systematic review of the marine natural-product literature identified 215 structures reported between July 2010 and August 2021 that were later found misassigned [20]. Erroneous NOE interpretation and comparison of NMR spectra each accounted for roughly 23% of cases. Total synthesis provided a definitive correction in 38% [20]. This subfield is far larger and more scrutinized than the eleven antibacterial cases reviewed here, yet its error rate persists. That scale marks false novelty as a structural feature of natural-product literature, not a lapse specific to antibacterials.

Eight independently documented cases from 2020 to 2025 (Table 3)—seven corrected misassignments and one originally unassigned stereochemistry later fixed by total synthesis—show that this failure mode is an active feature of the antibacterial literature, not a historical artifact. Each was resolved through convergent structural methods [21,22,23,24,25,26,27,28]. In six of the eight, the error or ambiguity was confined to stereochemistry rather than constitution. This concentration reflects a known asymmetry in analytical power: connectivity is reliably determined by routine spectra, whereas absolute configuration still depends on total synthesis or advanced computational methods such as ECD/TDDFT-ECD, DP4+, and DU8ML [9,22,29]. Two cases mark the pattern’s limit. Lydiamycin A [27] carried a constitutional error in its aliphatic acid side chain that survived an initial stereochemical revision until total synthesis exposed it. Halisphingosine A required a double-bond repositioning alongside its configurational revision [28]. Both show that verification must address the whole structure, not only the stereocenters flagged by a prior correction.

**Table 3:** Corrected structural misassignments and one originally unassigned stereochemistry were resolved by total synthesis. Extended version in Supplementary Table S2.

Compound	Source/Activity	Nature of Error	Verification Method
Myxovalargin [21]	Myxobacterial antibiotic; active vs. *M. tuberculosis* at sub-µg concentrations	Val7/Val10 D/L stereocenters reversed	Biosynthetic gene cluster analysis, total synthesis, cryo-EM of ribosome-bound complex
Chelocardin/amidochelocardin [22]	Atypical tetracyclines: active vs. Gram-negative ESKAPE pathogens	Absolute configuration misassigned	X-ray diffraction, NMR, ECD, TDDFT calculations
Serratiomycin [23]	Cyclic depsipeptide, known since 1998; new congeners active vs. *S. aureus* and *S. enterica*	L-Leu/L-allo-Thr/D-Ile corrected to D-Leu/L-Thr/L-allo-Ile	Marfey’s method, GITC derivatization, LC-MS, methanolysis, and modified Mosher’s method
Evybactin [24]	Depsipeptide; selectively active vs. *M. tuberculosis*	3-methylhistidine residue configuration misassigned	Total synthesis
Bacilotetrin A [25]	Cyclic lipodepsipeptide; anti-*Mycoplasma hyorhinis* activity, MIC 31 µg/mL (congeners)	Leucine/glutamate positions swapped in planar sequence	Reanalysis with newly reported congeners
Relacidine A/B [26]	Lipopeptides; active vs. colistin-resistant *E. coli*, MIC 2–4 µg/mL	Stereochemistry left unassigned since 2020 discovery—not an error, but an originally open gap, since resolved	Biosynthetic gene cluster analysis + bioinformatically guided total synthesis
Lydiamycin A [27]	Cyclodepsipeptide; antimycobacterial activity without cytotoxicity	Aliphatic acid moiety planar structure misassigned	Total synthesis; independently corroborated by bioinformatic, spectroscopic, and X-ray crystallographic analysis
Halisphingosine A [28]	Sphingoid base from marine sponge (*Haliclona* sp.); antibacterial, MIC 16–64 µg/mL vs. *E. coli* and 32–128 µg/mL vs. *S. aureus*	Relative and absolute configuration plus double-bond position revised to (2*R*,3*R*,8*R*,*Z*) from originally proposed structure	Total synthesis of 32 stereoisomers; NMR and chromatographic comparison

Two older total-synthesis cases extend the same failure mode over a longer horizon. Nicolaou et al. [30] found the reported structure of CJ-16,264 to be the wrong enantiomer entirely, and Jürjens et al. [31] resolved an analogous misassignment in the unrelated tetrapeptide GE81112A. Both predate the recent cohort; the 2020–2025 cases above establish misassignment as a live problem over the past five to six years.

The true cost of these misassignments extends well beyond a delayed correction to the record; it is opportunity cost in an already resource-constrained pipeline. When a misassigned structure with reported activity—such as myxovalargin [21] or evybactin [24]—enters the literature, it misdirects effort: synthetic chemists pursue incorrect scaffolds while medicinal chemists build SAR around a false pharmacophore. Given the prohibitive fully capitalized cost of antibacterial development [14], effort spent chasing such “phantom molecules” is drawn directly from genuine pipeline advancement.

This risk is distinct from rediscovery. A chemically known compound can, in principle, be excluded early through dereplication databases. A misassigned structure has no match for comparison and is undetectable by database comparison alone. It requires independent structural verification—a step that computational NMR methods such as DP4+ and DU8ML are designed to provide, but which de Albuquerque et al. [7] find is inconsistently applied. The eleven cases reviewed here comprise the eight structural misassignments of Table 3 and the three rediscoveries of Table 2. They are illustrative rather than exhaustive, but their recurrence across independent groups suggests uneven novelty-verification practices rather than isolated, unrelated anomalies.

Beyond its constitutional error, the halisphingosine A case also exposes how dereplication itself can fail at the analytical level. Sauer et al. [28] found the target and several synthetic regioisomers indistinguishable by mass spectrometry and chromatographic retention time alone, recommending co-injection spiking and NMR comparison to reliably differentiate them. This is direct evidence that a misassigned or rediscovered scaffold can survive routine MS-based dereplication undetected, precisely the failure mode this review identifies. The same failure recurs beyond the core set: the anti-MRSA metabolite verticilatin was synthesized in total [32], revising its originally proposed structure, and the macrolide tricholomenyn B was reclassified as a macrodiolide [33]. Both arise from fungal sources, reinforcing the idea that novelty-recognition failure is a general analytical problem rather than a taxon-specific artifact.

**Figure 1:**
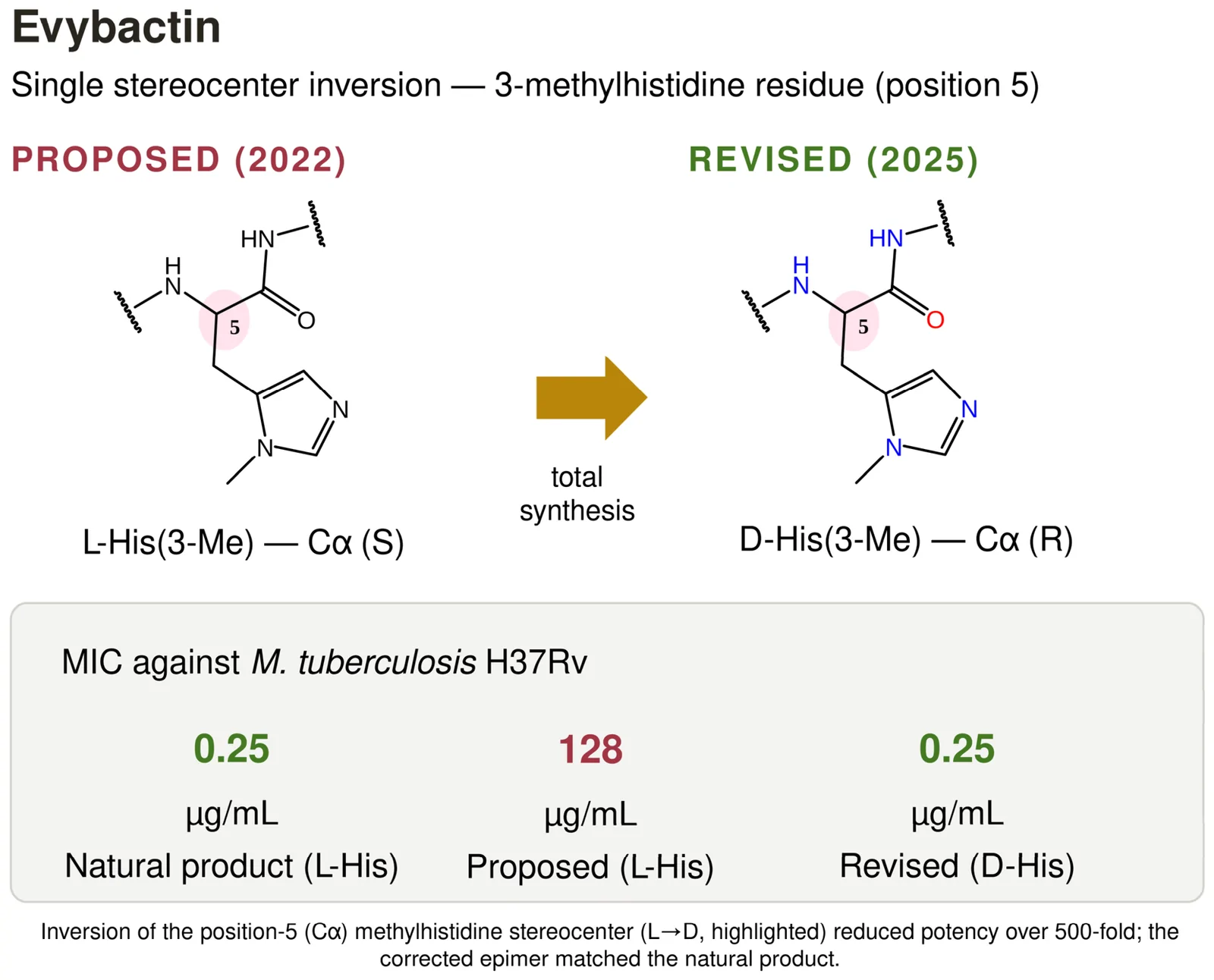
Single-stereocenter misassignment in evybactin. Inverting the position-5 stereocenter cut potency more than 500-fold against *M. tuberculosis*; the corrected epimer restored full activity [24].

**Figure 2:**
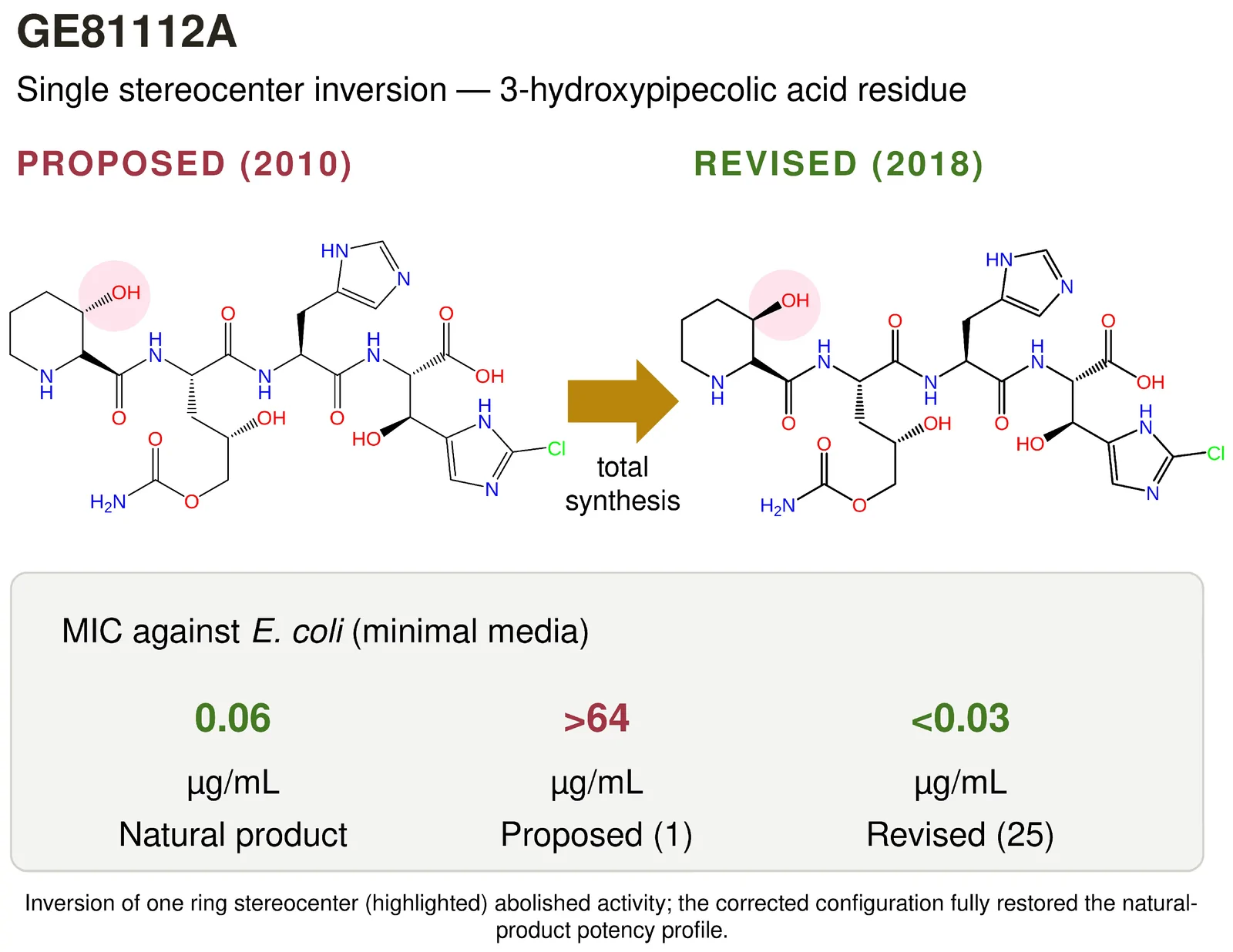
Single-stereocenter misassignment in GE81112A. Inverting one ring stereocenter abolished activity against *E. coli*; the corrected epimer matched the natural product’s potency [31]. GE81112A predates the 2020–2025 cohort and is shown here only to illustrate the mechanistic principle.

In most cases in Table 3, the opportunity-cost argument remains qualitative, as no direct comparison of bioactivity between the proposed and corrected stereoisomers has been reported [21]. Two cases permit a more concrete test because both were resolved only after the originally proposed structure was synthesized and found to be biologically silent. Lysenko et al. [24] found the proposed evybactin stereoisomer completely inactive against *M. tuberculosis*, with only the corrected epimer restoring activity (Figure 1). Jürjens et al. [31] report an analogous pattern for GE81112A, in which the proposed structure was inactive against *E. coli*, whereas the corrected epimer matched the natural product’s potency (Figure 2). In both cases, a single misassigned stereocenter separated a wholly inactive compound from a fully active one. Misassignment is therefore no mere bibliographic footnote; in some cases, it is a direct, measurable waste of synthetic and SAR effort.

This distinction shapes how the proposed framework must be structured. Dereplication addresses rediscovery, but misassignment requires a separate computationally assisted verification layer—one whose persistence through 2025 shows it is not yet standard in natural-product antibiotic discovery workflows [7,9].

## 5. Current Dereplication and Spectroscopic Practice: Capability Without Closure

If misassignment reflects a failure of interpretation, rediscovery reflects a failure of memory—and the natural-product field has spent the past decade building institutional memory into its workflows. The scale of this problem is not marginal: during antimicrobial screening, as many as 50% of actinomycete strains are discarded because they produce a known antibiotic [34]. That discard rate is the rediscovery tax the pipeline pays before isolation even begins, consuming screening capacity that scarce discovery-stage budgets can least absorb [14,34]. Wang et al. [35] formalized this effort by launching the Global Natural Products Social Molecular Networking platform (GNPS), a crowdsourced tandem mass spectrometry (MS/MS) reference library against which an extract can be screened before isolation begins. Novelty thereby becomes, in principle, a computable property of an MS/MS spectrum rather than a judgment reached only after weeks of purification. Yang et al. [36] had already established the underlying method—MS/MS molecular networking—clustering related metabolites by spectral similarity. This approach flags not only exact matches but also structural analogs, a meaningful advance over library searches that catch only identical compounds.

GNPS is not the only specialized resource built for this purpose. AntiBase compiles more than 100,000 of microbial, fungal, and algal metabolites with physicochemical and spectroscopic data for rapid dereplication [37]. MarinLit and the long-running annual “Marine natural products” review series serve an analogous role for marine-derived candidates [38]. Two further resources extend rather than duplicate this coverage. The Natural Products Atlas now catalogs more than 36,000 curated microbial natural products, along with structural reassignments, and has begun incorporating published corrections into the database itself [39]. The Dictionary of Natural Products offers the broadest cross-kingdom coverage, serving less as a rapid single-extract dereplication tool and more as an exhaustive check of the literature and history once a candidate structure is in hand [40].

Taken together, these resources make the recent rediscovery harder to attribute to a lack of reference infrastructure. They also make inconsistent adoption of existing infrastructure as one contributing factor in why rediscovery persists into the 2020s [5]. Yet the problem is not simply that the field has failed to use available tools. It is also that existing tools differ in what they can catch. Molecular networking excels at flagging compounds close enough to a reference spectrum to cluster with it. It is considerably less reliable at the network’s sparse periphery, where a genuinely novel compound and a poorly represented known compound can be visually and computationally indistinguishable. Sheng et al. [41] address this specific weakness directly. Their integrated molecular networking workflow (IMN4NPD) was built because standard molecular networking tends to prioritize larger clusters, overlooking self-looped or paired nodes—precisely the isolated, sparsely connected signals most likely to represent true novelty. Applied to an ethanol extract of *Plumula nelumbinis*, IMN4NPD surfaced novel compounds specifically among the self-looped nodes that standard networking had left unannotated [41]. That 2024 methods paper still frames this as an open problem, indicating that current dereplication workflows remain better optimized for confirming well-characterized compounds than for efficiently surfacing genuinely novel ones.

Misassignment, by contrast, is a defect of structure elucidation rather than of memory, so MS/MS matching cannot detect it. The relevant tool is quantum-chemical NMR calculation, such as DP4+, which compares a proposed structure’s predicted chemical shifts with the experimental spectrum using an explicit probability score rather than a qualitative “good fit” judgment [29]. Zhuang et al.’s [42] diphenazine work illustrates what this catches: three published structures were revised, and two new compounds were correctly characterized, precisely because empirical NOESY/ROESY analysis could not resolve several bridged stereocenters. Critically, this correction was achieved computationally rather than by total synthesis. It is a working model of verification applied before synthetic commitment—exactly the checkpoint this review argues should be routine [42]. Yet computational verification resolves stereochemical ambiguity more reliably than constitutional error; a wrong skeleton, as in lydiamycin A, it is less tractable to it beyond its reach, leaving total synthesis the final arbiter [19,27]. Its absence from routine practice is therefore an adoption problem, not a capability problem.

This asymmetry matters for two compounding reasons. The first is an incentive problem. GNPS-style dereplication is now a near-default first step [35,41]. DP4+-style verification [42], by contrast, remains a specialist intervention applied only once a structure has attracted enough biological or commercial interest to justify the effort. That ordering is backward, since the compounds most likely to receive rigorous re-verification are those already committed to downstream development. The second is a practical problem. Dereplication against a spectral library is comparatively cheap and fast. DP4+-style verification instead demands instrumentation access, computational literacy, conformational sampling, and statistical interpretation for each candidate structure, making it inherently harder to scale. The asymmetry is stark in terms of time. Acrosstwelve revised cases examined by Elyashberg et al. [43], computational reanalysis of the original published data resolved the correct structure within minutes—work that would otherwise have required full total-synthesis campaigns.

Structural correction is the scientific method working as intended. The concern is not that errors occur, but their timing: when correction follows synthesis rather than preceding it, self-correction becomes an expensive audit rather than an early filter.

The implication for pipeline renewal is precise rather than general. Further closing the rediscovery gap will require expanding library coverage and improving networking algorithms at the network’s sparse periphery [41]. Closing the misassignment gap requires something different: moving computational structure verification from a specialist, post hoc intervention to a default checkpoint applied before any bioactive natural product—particularly one with antibacterial activity—is reported as novel.

DP4+’s heavy computational demands limit its use as a high-throughput screen applied to every extract, creating room for a cheaper intermediate layer. Deep-learning models, particularly convolutional neural networks, increasingly automate dereplication and structural verification by replacing manual spectral comparison with pattern recognition. Tools such as the Small Molecule Accurate Recognition Technology (SMART) map uncharacterized metabolites against large chemical spaces directly from 2D NMR spectra such as HSQC [44,45]. Running on standard hardware without quantum-chemical calculations, this approach reduces the time and expertise costs of an initial novelty check sufficiently to apply it at the extract stage, before isolation. In the framework proposed below, the role of AI-based pattern recognition at Checkpoint 1 is a low-cost filter applied before the more expensive Checkpoint 3 verification is triggered. The section that follows develops this checkpoint-based logic into an explicit decision framework, specifying which check applies at each stage and at what cost.

## 6. Toward an Integrated Framework for Early Novelty Verification

Every resolution in Table 2 and Table 3—from Koller et al.’s [21] total-synthesis-confirmed revision of myxovalargin to Al Ayed et al.’s [26] bioinformatically guided total synthesis resolving the previously unassigned relacidine A/B stereochemistry—used a method already published and validated before the case was resolved. This confirms that persistence is a workflow integration and adoption problem rather than a tool development one, and it defines the kind of intervention this review can credibly propose. Table 4 summarizes the resulting diagnostic structure.

A useful diagnostic is to ask why these tools remain largely parallel rather than sequential in practice. Hu and Qiu’s [46] review of machine-learning-assisted structure annotation frames dereplication and structure elucidation as two stages of the same workflow. Yet the literature they survey treats them as separately optimized problems: one body of work refines library-matching algorithms for known-compound exclusion, while another refines quantum-chemical calculation for structure confirmation. Recent surveys of AI-assisted natural-product discovery reinforce this separation, cataloging dereplication and computational structure verification as distinct technique categories rather than as linked steps in a single novelty-verification workflow [47]. That survey inventories the individual tools without positioning any of them at a defined checkpoint that a compound must clear before a novelty claim is made. Few published workflows formally chain the two, such that a compound clearing dereplication automatically enters a verification step before that claim is advanced.

This reflects a deeper asymmetry in how the field allocates confidence. A compound that survives molecular networking without a spectral match earns provisional novelty by default. Yet the absence of a match is equally consistent with a poorly represented compound or an underpopulated database region [41], and such a compound typically proceeds to empirical structure elucidation with computational verification reserved for later. Verification is most valuable when a claim is cheapest to correct—before isolation, biological follow-up, or manuscript drafting—not after those investments are made.

Emerging machine-learning approaches to structure elucidation from spectral data offer a plausible route to narrowing this gap without requiring specialist quantum-chemistry expertise in every laboratory. Convolutional neural-network tools that annotate structures directly from NMR data illustrate how pattern recognition can accelerate structural hypothesis generation [45]. As these data-driven methods mature [46], the adoption barrier facing DP4+-style verification may narrow considerably in the coming years.

**Figure 3:**
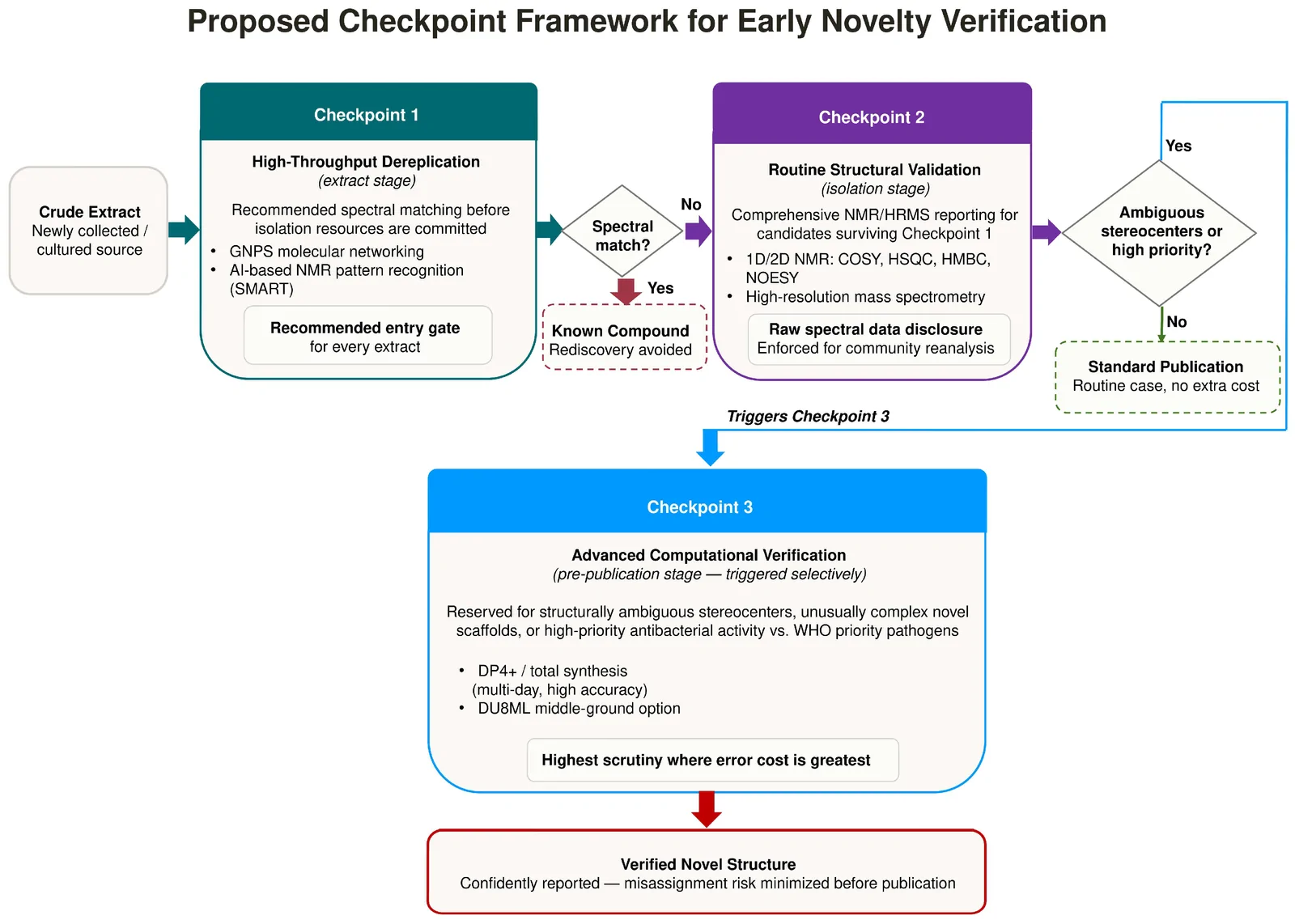
Proposed checkpoint-based framework for early novelty verification.

Recognizing that rigorous quantum-chemical verification for every isolated compound is economically and computationally unrealistic for many laboratories, this review proposes a checkpoint-based, resource-conscious workflow for early novelty verification, structured around three stages of the discovery pipeline (Figure 3). The proposal is a starting point rather than a finished protocol: its components draw on validated methods but have not yet been tested together as a single integrated pipeline.

Figure 3 summarizes the proposed Checkpoint 1–3 workflow and its trigger logic, while Supplementary Tables S1 and S2 map each reviewed case to its corresponding failure stage, downstream consequence, and preventable checkpoint. Critically, the three checkpoints are not redundant: each targets a distinct failure mode. Checkpoint 1 intercepts rediscovery, where a database match exists but was not sought. Misassignment carries no such match, so a genuinely novel but misinterpreted structure passes Checkpoint 1 by design and is instead caught downstream at Checkpoints 2 and 3, where the error is internal to the proposed structure itself. Myxovalargin is a case in point: its stereochemical discrepancy matched no database and was confirmed through total synthesis, complemented by cryo-EM of the ribosome-bound complex and resistant-mutant sequencing, tools characteristic of Checkpoint 3 [21].

Checkpoint 1: High-Throughput Dereplication (the extract stage). Before any isolation resources are committed, crude extracts should ideally undergo spectral matching using open-access mass spectrometry platforms such as GNPS molecular networking, coupled with emerging AI-based 1D/2D NMR pattern-recognition tools such as SMART [44,45]. SMART, for instance, was trained on over 2054 experimental HSQC spectra using a deep convolutional neural network that automatically learns spectral features, without investigator-defined rules [44]. This design allows such tools to deliver substantial cost and time savings over quantum-chemical verification, because they operate at the pattern-recognition level rather than the calculation level. This screening step is inexpensive relative to isolation. Treating it as a routine entry gate rather than an optional add-on would formalize a practice several groups already apply.

Checkpoint 2: Routine Structural Validation (the isolation stage). For candidates that survive Checkpoint 1, standard 1D and 2D NMR spectroscopy (COSY, HSQC, HMBC, NOESY), paired with HRMS, should be reported comprehensively.

Reliable structural validation, however, presupposes a chemically defined sample. Co-eluting impurities can introduce extraneous resonances that are misread as part of the target structure, making the misinterpretation of an impurity signal a documented source of error [48]. Reporting purity, ideally by quantitative NMR (qNMR), whose signal integrals are directly proportional to molar content, therefore provides a metrological anchor for the spectra on which all subsequent verification depends [49,50]. Coupling a stated purity value with open deposition of the underlying spectra enables reviewers to distinguish genuine structural features from sample-derived artifacts.

Journal guidelines could reinforce the inclusion of raw spectral data to enable community-driven reanalysis, a practice consistent with the broader push toward computational, data-driven structure verification [41,46].

Checkpoint 3: Advanced Computational Verification (the pre-publication stage). Resource-intensive methods such as DP4+, DU8ML, or total synthesis should not be required universally. Instead, Checkpoint 3 is reserved for candidates in which the downstream cost of error is greatest: structurally ambiguous stereocenters, unusually complex novel scaffolds, or high-priority antibacterial activity, as formally defined by WHO’s 2024 Bacterial Priority Pathogens List [51]. Rifampicin-resistant *Mycobacterium tuberculosis* sits in the highest-priority (critical) category of that list. Several anti-tuberculosis cases in Table 3, including myxovalargin, evybactin, and lydiamycin, therefore fall squarely within the critical category that would trigger Checkpoint 3.

To illustrate how this staged logic would operate in practice—without implying the framework has yet been validated as an integrated pipeline (see Section 8)—it is instructive to apply it retrospectively to cases already in Table 3. Evybactin’s misassigned configuration at the 3-methylhistidine residue, later corrected by total synthesis [24], sits squarely in the category that triggers Checkpoint 3, where configurational verification by computational NMR (DP4+/DU8ML) or synthesis would have been indicated before publication. Halisphingosine A illustrates the downstream cost of omitting this step: its revision required the total synthesis of 32 stereoisomers to resolve both configurational and double-bond assignments [28]. Conversely, lydiamycin A marks the boundary of what the framework can catch—its constitutional error in an aliphatic acid side chain survived an initial stereochemical revision until total synthesis exposed it [27], underscoring that Checkpoint 3 mitigates, but does not eliminate, misassignment risk.

Specifically for the computational option, DU8ML offers a practical middle ground between full DP4+ calculations and no verification at all. In a survey of nearly 170 alkaloids, its machine-learning-augmented DFT approach flagged 35 misassigned structures [52]. Across the reported cases, ^13^C chemical-shift deviations typically fell near 1 ppm, achieved far faster than full DP4+ analysis [53].

This staged approach also addresses the responsibility gap identified above. Beduru and Kutateladze’s [9] continuing catalog of misassigned structures through 2025 suggests limits in current review and reporting practices, particularly where raw spectral data and explicit verification statements are not routinely required. A field publishing under WHO’s documented pipeline scarcity [1] can ill afford structural corrections that arrive only after resources have already been committed downstream.

None of these checkpoints require new instrumentation or a fundamentally new discovery paradigm. What they require is reordering an existing toolkit around the specific failure modes traced in this review—rediscovery and misassignment. The intensity of verification is calibrated to the resource cost and biological stakes of each candidate. This replaces the current default of treating spectroscopic characterization as a single undifferentiated step positioned after bioactivity screening and before publication.

The feasibility of Checkpoint 3 should be acknowledged rather than assumed away. Beyond the computational turnaround noted above, DP4+-style analyses demand quantum-chemical software and expertise that are unevenly distributed across research groups and geographies. AI-assisted tools such as SMART partially offset this at Checkpoint 1 by automating pattern recognition before the more expensive quantum-chemical step is triggered [44,45]. These costs are plausibly justified by the downstream cost of an undetected misassignment, though a full economic accounting lies beyond the scope of this review. Lower-cost interim measures—such as journals requiring authors to state explicitly whether computational or synthetic verification was attempted and, if not, why—could narrow this gap without imposing a universal mandate.

Taken together, the framework addresses both failure modes described in this review through a single decision logic. Checkpoint 1 targets the memory failure that produces rediscovery, intercepting known compounds before isolation. Checkpoints 2 and 3 target the interpretation failure that leads to misassignment, testing whether a genuinely novel structure has been correctly read from its own spectra.

## 7. Driving Adoption

The checkpoint framework is only as effective as its uptake, and a recurring lesson from adjacent fields is that voluntary exhortation alone does not change practice. Within chemistry specifically, when a major publisher invited authors to deposit raw FAIR data on an optional basis, uptake remained low [54]. If complete structural verification is to become the norm rather than the exception, it must be embedded as a structural driver within the publishing and funding ecosystem rather than left to individual discretion.

Two mechanisms, each with an existing working precedent, can achieve this. The first is to make raw-data deposition a condition of publication. The most directly relevant precedent already exists within this discipline. Since June 2023, the Journal of Natural Products has required deposition of raw NMR data files in a public FAIR-compliant repository for all new compounds [55], moving beyond the older norm of supplying processed spectra alone. This requirement mirrors broader funder mandates such as the NIH Data Management and Sharing Policy, under which shared data should be made accessible no later than the time of an associated publication [56]. Mandatory deposition converts structural verification from a private step into a checkable public record. The second mechanism is to embed verification as an automated gate rather than an optional add-on. The strongest analog is the IUCr checkCIF system, which runs mandatory structure-validation tests on every submission. For Acta Crystallographica Sections C and E, unresolved alerts must be formally addressed during review [57]. An equivalent gate for natural-product structures—dereplication and, where warranted by risk, computational confirmation triggered within the discovery pipeline—would make verification the path of least resistance rather than an optional step.

Consistent with the checkpoint-based logic developed above, these drivers are calibrated to structural risk: not every compound requires total synthesis, but high-priority antibacterial scaffolds warrant the full checkpoint sequence. This preserves feasibility while directing the greatest verification effort toward the claims that carry the greatest consequence.

## 8. Limitations

This review is a critical narrative synthesis rather than an exhaustive census, and no formal quality scoring was applied. The eleven cases presented in Table 2 and Table 3 were selected for analytical relevance rather than comprehensive coverage [6,9]. Eleven cases, however well verified individually, cannot establish an incidence rate of false novelty across the natural-product antibacterial literature as a whole. The central claim is therefore qualitative, not causal. Misassignment and rediscovery persist despite instrumentation advances [7,41], but their precise share of pipeline attrition, relative to the economic and regulatory factors reviewed elsewhere [3], lies beyond what the available literature can support.

The case selection is also asymmetric in ways that warrant explicit acknowledgment rather than passing mention. The three rediscovery cases in Table 2 (2019–2022) are outnumbered by the eight cases in Table 3 (2020–2025), and the rediscovery cases skew earlier within the review’s overall window. This imbalance is consistent with the under-reporting argument made above—confirmed rediscoveries are less publishable and therefore harder to locate through literature search regardless of true incidence—but that explanation is itself unverified and cannot be distinguished, on the evidence assembled here, from a genuine difference in how often each failure mode occurs. Disconfirming evidence against the selection-bias account would take a specific form: a systematic re-screening of recent antibacterial natural-product publications (2020–2025) using dereplication databases such as GNPS or the Dictionary of Natural Products, applied uniformly regardless of whether the original authors reported a match, and finding that confirmed rediscoveries remain rare even under exhaustive search. No such systematic re-screening has been conducted here or, to my knowledge, published elsewhere, so this possibility remains open. The eleven-case sample is best read as a demonstration that both failure modes occur and persist, not as a basis for comparing their relative frequency.

A further limitation concerns the proposed framework itself: although each checkpoint draws on individually validated methods, the three-checkpoint workflow has not been tested prospectively as a single integrated pipeline. It is therefore best regarded as a conceptual proposal requiring prospective validation, not a protocol demonstrated to reduce false novelty in practice.

Three biases may further shape case selection and interpretation. Publication bias likely suppresses confirmed rediscoveries, reinforcing the under-reporting reading above. Citation bias may favor prominent misassignment corrections over quieter rediscovery reports. Database bias matters because dereplication depends on reference coverage, so compounds from under-represented taxa may be misjudged as novel. These constraints are acknowledged, not eliminated.

## 9. Conclusions

The eleven cases reviewed here illustrate that early novelty-recognition failure remains a current and methodologically consequential problem in antibacterial natural-product research, even if its prevalence cannot yet be quantified. Existing dereplication and verification tools, therefore, matter not as future possibilities but as underintegrated safeguards whose absence carries a real opportunity cost under conditions of pipeline scarcity. Correcting this problem will not, by itself, solve antibacterial pipeline attrition. It will, however, improve the odds that scarce downstream resources are spent on candidates whose novelty has been tested earlier, more proportionately, and with greater procedural consistency.

## Figures and Tables

**Table 1:** Databases, representative search terms, and targeted publication range.

Database/Source	Representative Search Terms	Publication Range Targeted
PubMed and Web of Science	(natural product) AND (misassign* OR reassign* OR (revis* AND structure)); antibacterial AND (rediscover* OR reisolat* OR dereplicat*); “total synthesis” AND (structur* AND revis*)	2019–2025
Dictionary of Natural Products	Targeted compound-name and molecular-formula lookups for candidate rediscovery/misassignment cases identified via PubMed/Web of Science	Full database (no date restriction; used for dereplication, not primary discovery)
Citation tracking (forward/backward)	Citing and cited references of Yoo et al. [6] and Beduru and Kutateladze [9]	No date restriction; captures earlier and more recent structure-revision literature

* Truncation (wildcard) operator retrieving all variant word endings (e.g., dereplicat* → *dereplicate, dereplication*).

**Table 4:** Two novelty-recognition failure modes and their established technical remedies.

Novelty-Recognition Failure	Underlying Mechanism	Detection Signature	Established Technical Remedy
Rediscovery	Failure of institutional memory: a bioassay-guided hit matches an already-known metabolite that was not checked against existing spectral records	Positive match against a reference spectral library or database	Molecular networking/GNPS-based dereplication [35,36,41]
Structural misassignment	Failure of interpretation: incomplete or ambiguous spectroscopic evidence supports an incorrect structure for a genuinely unfamiliar compound	No database match exists to search against; the error is internal to the proposed structure itself	Computational NMR structure verification: DP4+, DU8ML [9,29]

## Data Availability

No new data were generated in this review. All case data discussed are drawn from the published literature cited in the References section and in Supplementary Tables S1 and S2.

## Supplementary Materials

The following supporting information can be downloaded at: https://www.mdpi.com/article/10.3390/ph19081182/s1. Figure S1: Qualitative case-selection flow. Table S1: Verified rediscovery cases with failure-stage and checkpoint annotations. Table S2: Corrected structural misassignments and one originally unassigned stereochemistry, with failure-stage and checkpoint annotations.

## Abbreviations

The following abbreviations are used in this manuscript:

AMR	Antimicrobial resistance
NMR	Nuclear magnetic resonance
2D NMR	Two-dimensional nuclear magnetic resonance
HSQC	Heteronuclear single quantum coherence
HMBC	Heteronuclear multiple bond correlation
COSY	Correlation spectroscopy
NOESY	Nuclear Overhauser effect spectroscopy
ROESY	Rotating-frame Overhauser effect spectroscopy
MS	Mass spectrometry
HRMS	High-resolution mass spectrometry
LC-HRMS	Liquid chromatography–high-resolution mass spectrometry
ECD	Electronic circular dichroism
DFT	Density functional theory
TDDFT	Time-dependent density functional theory
DP4+	DP4+ probability (NMR-based structure verification)
DU8ML	DU8ML (machine-learning NMR prediction method)
GNPS	Global Natural Products Social Molecular Networking
SMART	Small Molecule Accurate Recognition Technology
MIC	Minimum inhibitory concentration
SAR	Structure–activity relationship
WHO	World Health Organization
FID	Free induction decay
FAIR	Findable, Accessible, Interoperable, Reusable (data principles)
NIH	National Institutes of Health
