# Supplementary material for: False Novelty in Antibacterial Natural-Product Discovery: Rediscovery, Structural Misassignment, and a Checkpoint Framework for Early Verification
Source: Pharmaceuticals (Basel). 2026 Jul 28;19(8):1182. doi: 10.3390/ph19081182 (PMC13516265; doi:10.3390/ph19081182)
Supplement: Supplementary file 1 [file pharmaceuticals-19-01182-s001.zip › pharmaceuticals-4450571-supplementary.pdf]

Supplementary Data

Figure S1. Qualitative case-selection flow

Table S1. Verified rediscovery cases with failure-stage and checkpoint annotations.

Table S2. Corrected structural misassignments and one originally unassigned stereochemistry, with failure-stage and checkpoint annotations.

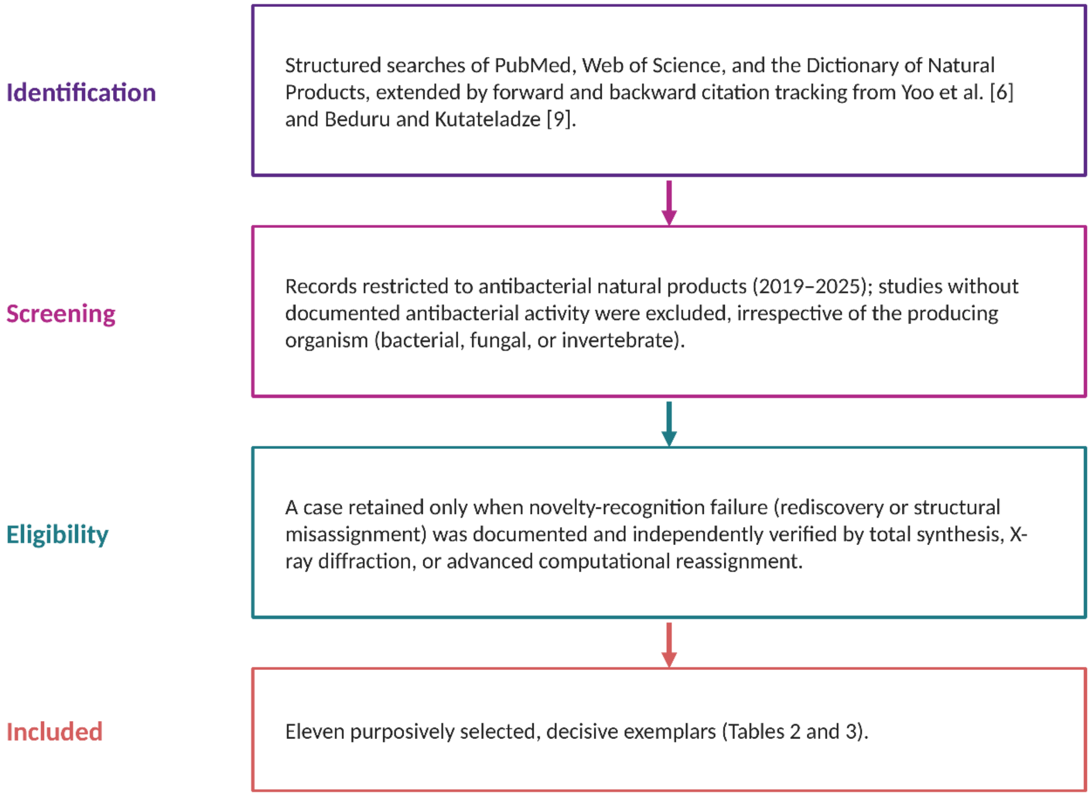

Figure S1. Qualitative case-selection flow.

The two tables below reproduce Tables 2 and 3 with additional columns — failure stage, downstream consequence, preventable checkpoint, and why the case matters — mapping each case explicitly onto the checkpoint-based framework proposed in this review. They are presented here in landscape orientation for readability and are referenced in the main text as Table S1 and Table S2.

Table S1. Verified rediscovery cases with failure-stage and checkpoint annotations.

Compound Pair	Original / Later Identity	How Identity Was Established	Failure Stage	Downstream Consequence	Preventable Checkpoint	Why This Case Matters	Ref.
Cottoquinazoline F	Already reported from an arthropod-derived strain of *N. fischeri*; reisolated during an unrelated stereochemical study	Reisolation and configurational reinvestigation; antibacterial activity vs. ESBL-producing *E. coli* and others recognized only incidentally	Post-isolation (identity recognized only after full characterization)	Isolation and structure-elucidation resources were spent on an already-known metabolite before the duplication was recognized	Pre-isolation dereplication against public spectral/genomic databases (Checkpoint 1) would likely have flagged the match at the crude-extract stage	Activity alone does not establish novelty; the antibacterial signal was real but attached to a known scaffold	[10]
Triculamin = Alboverticillin	Triculamin (isolated 1967) shown identical to alboverticillin (isolated 1958); two names for one lasso peptide for over 50 years	2D NMR spectroscopy combined with genome sequencing during biosynthesis investigation	Long-latent identity gap (decades between original reports and recognition)	Two independent literatures accumulated around the same molecule under different names, fragmenting the evidence base	Cross-referencing new isolates against legacy natural-product literature, not only recent databases, before assigning a new name	Rediscovery risk extends to older literature not indexed in modern spectral databases	[11]
Leepeptin / Chaxapeptin	Of three lasso peptides from *S. leeuwenhoekii* C34^{T}, one novel (leepeptin); the other two matched known peptides—chaxapeptin (2025, Lp3) and a,citrulassin-family peptide (Lp1)	LC-HRMS and NMR comparison within the same heterologous expression study	Within-study triage (identity resolved before external publication)	Limited downstream consequence; the case demonstrates the discriminating power of paired LC-HRMS/NMR when applied early	Routine Checkpoint 1-style practice already applied here; included as a positive control showing early screening working as intended	Systematic within-study dereplication can catch rediscovery before it reaches the literature	[12]

Table S2. Corrected structural misassignments and one originally unassigned stereochemistry, with failure-stage and checkpoint annotations.

Compound	Source / Activity	Nature of Error	Verification Method	Failure Stage	Downstream Consequence	Preventable Checkpoint	Why This Case Matters	Ref.
Myxovalargin	Myxobacterial antibiotic; active vs. *M. tuberculosis* at sub-µg concentrations	Val7/Val10 D/L stereocenters reversed	Biosynthetic gene cluster analysis, total synthesis, cryo-EM of ribosome-bound complex	Pre-publication (error present at original structure report)	Mechanism studies were built on an incorrect stereochemical model until cryo-EM and total synthesis corrected it	Routine Checkpoint 3 verification before publication, given sub-µg activity	Misassignment can persist into mechanism-of-action studies, not just the structural literature	[21]
Chelocardin / amidochelocardin	Atypical tetracyclines; active vs. Gram-negative ESKAPE pathogens	Absolute configuration misassigned	X-ray diffraction, NMR, ECD, TDDFT calculations	Pre-publication	Gram-negative ESKAPE activity data were associated with an incorrect absolute configuration for an extended period	ECD/TDDFT-based configurational check (Checkpoint 3) triggered by priority-pathogen activity	Priority-pathogen activity is exactly the profile the review's Checkpoint 3 criteria are designed to capture	[22]
Serratiomycin	Cyclic depsipeptide, known since 1998; new	L-Leu/L-allo-Thr/D-Ile corrected to D-Leu/L-Thr/L-allo-Ile	Marfey's method, GITC derivatization, LC-MS,	Long-latent (parent structure	A known scaffold's stereochemistry remained wrong in the literature for	Periodic re-verification of legacy structures when	Even long-published, seemingly settled structures are not	[23]

Compound	Source / Activity	Nature of Error	Verification Method	Failure Stage	Downstream Consequence	Preventable Checkpoint	Why This Case Matters	Ref.
	congeners active vs. *S. aureus* and *S. enterica*		methanolysis, modified Mosher's method	unresolved for over two decades)	about 20 years before new congeners prompted re-examination	new congeners or activity data reopen a scaffold	immune to misassignment	
Evybactin	Depsipeptide; selectively active vs. *M. tuberculosis*	3-methylhistidine residue configuration misassigned	Total synthesis	Pre-publication	Selective anti-tubercular activity was attributed to a structure later shown to require full resynthesis for confirmation	Total synthesis or DP4+ verification (Checkpoint 3) for a single ambiguous stereocenter before publication of a selectively active candidate	A single misassigned stereocenter can alter biological interpretation for a high-priority pathogen target	[24]
Bacilotetrin A	Cyclic lipodepsipeptide; anti-*Mycoplasma hyorhinis* activity, MIC 31 μg/mL (congeners)	Leucine/glutamate positions swapped in planar sequence	Reanalysis with newly reported congeners	Pre-publication (constitutional, not stereochemical)	Anti-*Mycoplasma* activity data were associated with an incorrect planar sequence until congener discovery prompted reanalysis	Cross-checking planar sequence against related congeners at the point of initial characterization (Checkpoint 2)	One of the few constitutional, not merely stereochemical, errors in the set; Checkpoint 2 rigor also matters	[25]
Relacidine A / B	Lipopeptides; active vs. colistin-resistant *E. coli*, MIC 2–4 μg/mL	Stereochemistry left unassigned since 2020 discovery — not an error, but an originally open gap, since resolved	Biosynthetic gene cluster analysis + bioinformatically guided total synthesis	Unresolved gap (multi-year, not a misassignment per se)	A candidate active against colistin-resistant Gram-negative bacteria carried unresolved stereochemistry for several years	Mandatory stereochemical resolution (Checkpoint 3) before an unusually active candidate advances further in the pipeline	An unresolved gap can carry downstream risk similar to an outright misassignment	[26]
Lydiamycin A	Cyclodepsipeptide; antimycobacterial activity without cytotoxicity	Aliphatic acid moiety planar structure misassigned	Total synthesis; independently corroborated by bioinformatic, spectroscopic, and X-ray crystallographic analysis	Post-correction residual error (missed even after an initial stereochemical revision)	A constitutional error persisted through an initial correction cycle before total synthesis exposed the remaining error	Verification protocols addressing the whole structure, not only the stereocenters flagged by a prior correction	Partial correction can leave consequential errors undetected	[27]
Halisphingosine A	Sphingoid base from marine sponge (*Haliclona* sp.); antibacterial, MIC 16–64 μg/mL vs. *E. coli* and 32–128 μg/mL vs. *S. aureus*	Relative and absolute configuration plus double-bond position revised to (2R,3R,8R,Z) from originally proposed structure	Total synthesis of 32 stereoisomers; NMR and chromatographic comparison	Pre-publication (constitutional plus configurational error)	The proposed structure was indistinguishable from synthetic regioisomers by MS and retention time alone, allowing the error to survive routine dereplication	Independent structural verification via NMR comparison and computational methods (DP4+/DU8ML, Checkpoint 3); co-injection spiking exposes the Checkpoint 1 blind spot	Demonstrates that a misassigned scaffold can survive MS-based dereplication undetected, and that verification must address the whole structure, not only stereocenters	[28]
